# Ultrafine particles altered gut microbial population and metabolic profiles in a sex-specific manner in an obese mouse model

**DOI:** 10.1038/s41598-021-85784-4

**Published:** 2021-03-25

**Authors:** Kundi Yang, Mengyang Xu, Jingyi Cao, Qi Zhu, Monica Rahman, Britt A. Holmén, Naomi K. Fukagawa, Jiangjiang Zhu

**Affiliations:** 1 Department of Chemistry and Biochemistry, Miami University, Oxford, OH 45056 USA; 2 Department of Biology, Miami University, Oxford, OH 45056 USA; 3 School of Engineering, University of Vermont, Burlington, VT 05405 USA; 4 USDA ARS Beltsville Human Nutrition Research Center, Beltsville, MD 20705 USA; 5 Department of Human Sciences, The Ohio State University, 302D Wiseman Hall, 400 W 12th Ave, Columbus, OH 43210 USA; 6 James Comprehensive Cancer Center, The Ohio State University, Columbus, OH 43210 USA

**Keywords:** Environmental impact, Biochemistry, Biotechnology, Microbiology, Environmental sciences, Biomarkers, Analytical chemistry

## Abstract

Emerging evidence has highlighted the connection between exposure to air pollution and the increased risk of obesity, metabolic syndrome, and comorbidities. Given the recent interest in studying the effects of ultrafine particle (UFP) on the health of obese individuals, this study examined the effects of gastrointestinal UFP exposure on gut microbial composition and metabolic function using an in vivo murine model of obesity in both sexes. UFPs generated from light-duty diesel engine combustion of petrodiesel (B0) and a petrodiesel/biodiesel fuel blend (80:20 v/v, B20) were administered orally. Multi-omics approaches, including liquid chromatography–mass spectrometry (LC–MS) based targeted metabolomics and 16S rRNA gene sequence analysis, semi-quantitatively compared the effects of 10-day UFP exposures on obese C57B6 mouse gut microbial population, changes in diversity and community function compared to a phosphate buffer solution (PBS) control group. Our results show that sex-specific differences in the gut microbial population in response to UFP exposure can be observed, as UFPs appear to have a differential impact on several bacterial families in males and females. Meanwhile, the alteration of seventy-five metabolites from the gut microbial metabolome varied significantly (ANOVA *p* < 0.05) across the PBS control, B0, and B20 groups. Multivariate analyses revealed that the fuel-type specific disruption to the microbial metabolome was observed in both sexes, with stronger disruptive effects found in females in comparison to male obese mice. Metabolic signatures of bacterial cellular oxidative stress, such as the decreased concentration of nucleotides and lipids and increased concentrations of carbohydrate, energy, and vitamin metabolites were detected. Furthermore, blood metabolites from the obese mice were differentially affected by the fuel types used to generate the UFPs (B0 vs. B20).

## Introduction

Obesity has been reported to associate with diabetes, atherosclerosis and hypertension^1^, and approximately sixty-five percent of adults in the US, and more than a hundred billion people worldwide, have been diagnosed as overweight or obese^2^. Substantial epidemiologic evidence also suggests that obesity causes a state of chronic subclinical inflammation, which mediates most of the systemic complications associated with obesity^3^. Obesity is typically accompanied by metabolic problems such as blood pressure elevation, insulin resistance, and atherogenic dyslipidemia, and these problems often contribute to obesity-related tissue injury^4^. Further, it is increasingly recognized that environmental factors such as air pollution may contribute to the global obesity epidemic, and epidemiologic studies have shown an association between air pollution and obesity-related comorbidities^5,6^.

Particulate matter (PM), a component of air pollution, is ubiquitous in indoor and outdoor environments. Many situations and activities performed by the general population on a regular basis, such as urban-dwelling, driving, and outdoor exercise can lead to significant human UFP exposure. Based on epidemiological evidence, exposure to elevated concentrations of ambient particulate matter with an aerodynamic diameter less than 10 µm and 2.5 µm (PM_10_ and PM_2.5_) is associated with increased pulmonary and cardiovascular diseases^7,8^, inflammation, as well as obesity and its associated morbidity and mortality^9,10^. However, a considerable knowledge gap about the health effects of particles less than 100 nm, known as ultrafine particles (UFPs), exists. Increasingly, recent studies provided evidence that these submicron-scale particles exhibit significantly different physicochemical properties than the larger PM, and UFPs cause adverse health effects through different mechanisms^11^.

One of the most important sources of PM in ambient air is diesel engine exhaust^12^. Since 2007, significant technological improvements in engine and pollution control designs for diesel engines, including the use of “cleaner” fuels, have lowered regulated exhaust emissions (i.e., PM, NOx, and CO), but the unregulated UFPs are still produced and pose an important human health risk^13–15^. Altering diesel fuel composition is one approach to reduce toxic and net greenhouse gas emissions as well as increase domestic energy security. Blending petrodiesel with biodiesel (a mixture of fatty acid methyl esters, FAMEs, derived from animal fats or vegetable oils via transesterification) is now widespread due to biodiesel’s commercial availability. In the U.S., commonly-used commercial biodiesel fuel is derived from soybean oil and blended with petrodiesel up to 20% v/v biodiesel (B20), but FAMES is allowed up to 5% v/v in “diesel” fuel, meaning biodiesel combustion products are widespread, despite relatively few studies on the composition of exhaust biodiesel PM^16,17^. In fact, lower amounts of CO, PM mass, and polycyclic aromatic hydrocarbons (PAH) are emitted when biodiesel is used compared to petrodiesel fuel^18,19^. The underlying mechanism contributing to the adverse health effects of PM exposure is thought to be related to the production of reactive oxygen species (ROS) or via ROS-bearing functionalities within the particles^20,21^. However, there is still debate about whether biodiesel UFPs can induce inflammation more readily than petrodiesel UFPs. Some researchers have suggested that the higher levels of polar organic compounds in biodiesel, as compared to petrodiesel, results in higher ROS production^22^. For example, lung cell studies have suggested that enhanced cytotoxicity and inflammatory responses can be induced by biodiesel compared to petrodiesel^23^. In contrast, other investigators have reported reduced ROS production and DNA damage for biodiesel compared to petrodiesel^13^.

Inhalation of UFPs is considered to be the primary exposure path for humans, making the lungs a primary target. However, the gastrointestinal tract is also directly impacted by UFPs^24^. The intestine can be exposed to inhaled UFPs by mucociliary transport from the lung to other organs, including the gastrointestinal tract^25–27^. There are reports that UFPs can contribute to gastrointestinal diseases such as inflammatory bowel disease (IBD)^28,29^. Potential mechanisms for UFP ingestion to cause gastrointestinal diseases to include immune activation, systemic inflammation, and intestinal microbiota modulation induced by UFPs exposure, which consequentially impacts the gut epithelial cells^25^. Although recent studies suggested that UFP exposure may be associated with inflammatory bowel disease and its associated comorbidities, there is still a lack of clear evidence about how UFPs affect the metabolism of gut microbiota which may subsequently adversely affect the host’s metabolic health. Further, while many metabolic disorders, characterized by low-grade inflammation, are associated with obesity^30,31^, recent studies suggest that exposure to traffic-related air pollution, including UFPs, may play an important role in the development and exacerbation of metabolic diseases^32–34^. Evidence shows that gut microbiota can be different among lean, obese, and type 2 diabetic patients and this difference may play an important role in our understanding of the pathogenesis of metabolic diseases^35,36^.

Previous studies have demonstrated that the microbiota of obese animals is more efficient at extracting energy from a given diet than the microbiota of lean animals^37^, which suggests a greater efficiency of the obese host metabolism, and highlights the need to specifically studying the impact of UFPs on obese subjects. In the current study, we examine the effects of UFP on the gut microbiome of obese animal models to test the hypothesis that UFP generated from the combustion of petrodiesel versus biodiesel fuel may exacerbate gut microbial dysbiosis and metabolic dysfunctions related to obesity. Our primary objectives were to determine whether the exposure to UFP generated from the combustion of two diesel fuels differentially alters (1) the gut microbiota community of the obese rodent, (2) the gut metabolic profiles, and (3) host metabolism with a fuel-type specific effect. A high-fat diet-induced obese mouse model was used in this study to compare exposure to phosphate buffer solution (PBS) as controls to two different types of UFPs to determine the effect of UFPs in obese subjects. Furthermore, we used both female and male mice to investigate whether there are sex-specific responses in obese mice after exposure to UFPs. Liquid chromatography-mass spectrometry (LC–MS) targeted metabolomics of cecal and plasma samples, and 16S rRNA gene sequencing analyses of mouse cecal samples were performed and the metabolites that potentially contribute to the connection between the intestinal microbiome and host metabolome were evaluated. The study outcomes lay the groundwork for future mechanistic investigations of increased risk of metabolic dysfunction in obese individuals.

## Materials and methods

### Mouse husbandry, exposure to UFP and sample collection

The detailed ultrafine particle generation, collection, and characterization procedures were provided in our previous publications^20,38^. Briefly, UFPs were generated, collected, concentrated and characterized by the University of Vermont Transportation Air Quality laboratory, and the particle stock suspension was prepared at the final concentration of 0.35 mg/mL before the oral administration to the tested animals. Three-week-old male and female C57B6 specific pathogen-free mice were purchased from the Jackson Lab (Bar Harbor, ME, USA), and housed in a clean, pathogen-free environment under controlled lighting (12 h light: dark; lights on at 6 a.m. and lights off at 6 p.m.) and temperature (21 °C). The animals were housed in pairs for 4 weeks and then housed individually for the rest of the experiment. The animal experimental protocol was approved by the Institutional Animal Care and Use Committee (IACUC) at Miami University following the National Institutes of Health Guidelines for the Care and Use of Laboratory Animals, and is in compliance with the ARRIVE guidelines^39^. After power analysis and reference to our earlier study^40^, a total of 36 mice (18 males and 18 females) were divided into 6 groups (3 groups of n = 6 for each group) to establish the obese mice model. After a week of acclimation, the thirty-six 4-weeks old mice were fed a high-fat diet (45 kcal% Fat, Research Diets, Inc Indianapolis, Indiana, USA) for another six weeks to induce the obese phenotype. Weight difference monitoring (weekly) in control groups of n = 6 mice for each sex fed a normal diet (10 kcal% Fat, Research Diets, Inc Indianapolis, Indiana, USA) confirmed that the experimental protocols resulted in significant body weight increase in the high-fat diet animals (*p* = 0.012 for male groups and *p* = 1.16 E−05 for the female groups) compared to six normal chow-fed mice for each sex. After the confirmation, only the obese mice groups were used in further experiments. All obese mice used in this experiment were at the age of ~ 10 weeks when treatments were initiated. Three groups of obese mice from each sex were treated with PBS (serve as vehicle control), B0 and B20 at the concentration of 0.35 mg/mL by oral gavage without sedation to achieve precise enteric administration; 2 mL of each suspension (~ 0.70 mg UFP mass) was administered to the mice every other day for a total of 10 days (i.e., 5 administrations per mouse for a total of 10 mL of the suspension over 10 days)^41^. Body weight was measured again at the time of harvest, and no significant body weight differences were observed among the PBS, B0, and B20 groups within each sex. Mice were anesthetized by isoflurane followed by decapitation after the 10-day treatment period. The whole blood sample was collected in a 2 mL tube containing 10µL heparin, placed on ice, centrifuged and the obtained plasma transferred into a new 2 mL centrifuge tube then immediately transferred to − 80 °C freezer for long term storage. Cecal contents were collected on ice and immediately stored at − 80 °C until further analyses.

### DNA isolation and 16S ribosomal DNA sequencing

The 16S ribosomal DNA sequencing was performed for metagenome analysis by following a modified version of an established protocol published previously. DNA extraction from cecal content was performed using the Fast DNA Spin Kit for Feces (MP Biomedicals). Briefly, 500 mg of caecal samples were mixed with 825 µL of sodium phosphate buffer, 122 µL of MT buffer, and 0.5 mL of 0.1 mm zirconia/silica beads (BioSpec Products, Bartlesville, OK, USA). Samples were homogenized with a bead beater (BioSpec Products, Bartlesville, OK, USA) for 3 min at a setting of 6.0 m/s for 40 s. The DNA recovered from these samples was assessed using NanoDrop and gel electrophoresis. Purified genome DNA samples were sent to the Miami University Center for Bioinformatics and Functional Genomics (CBFG) for 16S rDNA sequencing. 515f/806r primer set that the EMP (Earth Microbiome Project) (http://gilbertlab.com/contact-us/) pioneered and the GoTaq Hot Start Colorless Master Mix (Promega) was used for PCR amplification of the 16S rDNA V4 region. Each DNA sample was amplified using a reverse primer tagged individually with a unique 12-base Golay barcode. Agarose gel electrophoresis was conducted for the quality check of PCR products. Amplified 16S rDNA library and Amplicons were purified by the SequalPrep Normalization Plate kit (Thermo Fisher, Waltham, MA, USA). Purified products were quantified by KAPA Library Quantification Kit Illumina Platforms (Kapa Biosystems, Wilmington, MA, USA). An Illumina Next Generation Sequencing (NGS) MiSeq platform was used for amplicon sequencing.

### Bioinformatics analysis of genomic data

Illumina generated fastq files were demultiplexed, processed, quality filtered, and analyzed by Quantitative Insights into Microbial Ecology (QIIME, V 1.9.1, and V 2018.4)^42^. The DADA2 software package in QIIME 2^43^ was used for denoising paired-end Illumina sequenced fastq files, dereplicating them, and filtering chimeras. Operational Taxonomic Unit (OTU) and taxonomic binning of classified sequences were obtained based on a pre-trained SILVA database (version 128) at 97% sequence similarity^44^ for the taxonomic classification of the sequence reads by using the feature classifier plugin^45^, and OTUs were identified from the sequence results to assign the relative abundance of bacteria at different taxonomic levels. The number of sequences varied between samples, ranging from 3130 to 84,298 with a mean number of 37,534 (median: 37,382). The 4000 threshold was chosen according to the rarefaction curve (Figure S1), at which point the rarefaction curve tended to be stable and therefore indicates a good representation of the microbial community. The samples exhibiting a limited number of sequences (less than 4000) were therefore excluded from the study. In addition, the QIIME2 taxa bar plot command was used for viewing the taxonomic composition of the samples. A QIIME 2 plugin, longitudinal, was performed for beta-diversity analyses^46^. Principal coordinate analyses (PCoA) were performed based on Jaccard distances in QIIME2. Beta diversity results were scrutinized using principal coordinate analysis (PCoA) that measured bacterial community relatedness, which represented the variation in species composition among different treatment groups^47^. To detect statistical differences in beta diversity metrics between groups, group significance test and visualization of beta diversity parameters were carried out using permutational Multivariate Analysis of Variance (PERMANOVA) with the ‘Qiime diversity beta-group-significance’ plugin^48^. A pairwise significance test was also performed comparing groups using the same distance matrix metrics. Differential abundance of taxa between UFP treated groups (B0 and B20) and PBS groups was determined at the family level using the *DESeq2* package in R^49^.

Phylogenetic Investigation of Communities by Reconstruction of Unobserved States (PICRUST, Galaxy V1.1.1) was used to predict microbial function^50^. A closed-reference OTU-picking strategy in QIIME 1 was used for assigning the sequencing reads to species equivalent operational taxonomic units (OTUs) (at 97% sequence similarity) with GreenGenes (gg_13_08)^51^. The output OTU table was normalized to 16S rRNA gene copy number from known bacterial genomes in Integrated Microbial Genomes. Gene function was then predicted based on the Kyoto Encyclopedia of Genes and Genomes (KEGG) database.

### Metabolite extraction

Cecal sample metabolite extraction was performed using a modified cold methanol extraction method. Briefly, pellets from each mouse cecal sample were weighed and recorded for use in normalization procedures. The weighed cecal samples (15–20 mg) were homogenized with 500 µL PBS solution, followed by the addition of 500 µL cold MeOH and mixing. The mixture was then quenched at − 20 °C for at least 20 min followed by the addition of 50 µL isotope-labeled amino acid mixture as internal standards (Cambridge Isotope Laboratories, Inc.). The samples were vortexed vigorously for 1 min and 450 µL of extracted supernatant collected after centrifugation at 14,000 rpm for 5 min and then dried in a vacuum concentrator. A mixture of 50% acetonitrile combined with 50% ultrapure water was used to reconstitute the sample. The final samples were loaded into liquid chromatography vials for analysis.

### Metabolic profiling

The targeted metabolic profiling method used in this study is similar to our previous work^52–54^. Retention time and selected reaction monitoring (SRM) transition of targeted metabolites were established by running pure standards (purchased from Sigma, Saint Louis, MO, USA and IROA Technology, Boston, MA) and collecting the tandem mass spectrum (MS/MS), so the orthogonal information of retention time and two pairs of SRM transitions could be used to confidently detect and identify targeted compounds. Briefly, a Thermo Scientific TSQ Quantiva triple quadrupole mass spectrometer equipped with an electrospray ionization source was applied to both positive and negative mode compound detection, which coupled with a Thermo Scientific Ultimate 3000 high-performance liquid chromatography (HPLC) equipped with a hydrophilic interaction chromatography (HILIC) column (Waters Corporation, Milford, MA, USA, 2.1 × 150 mm, amide 2.5 µm). The auto-sampler temperature was kept at 4 °C, the column compartment was set at 40 °C, and the separation time for each sample was 20 min. The reconstituted cecal samples were gradient-eluted at 0.300 mL/min using solvents A (5 mM ammonium acetate in 90% water/10% acetonitrile + 0.2% acetic acid) and B (5 mM ammonium acetate in 90% acetonitrile/10% water + 0.2% acetic acid). Stable ^13^C and ^15^N universal labeled amino acid mix (Cambridge Isotope, Tewksbury, MA) were used as internal standards for quality control purposes. Pooled quality control samples were included after ten samples were run to monitor instrument stability.

### Statistical analyses

The data were analyzed by Statistical Analysis of Metagenomic Profiles (STAMP, V2.1.3) with removed unclassified reads. Welch’s t-test with a *p* value filter of *p* ≤ 0.05 was used to identify the differences between groups. The Quanbrowser module of Xcalibur 4.0 was used to manually process targeted metabolite profiling data. Acquired peak intensities were normalized by the weight of cecum samples. MetaboAnalyst 4.0 was used for statistical analysis of metabolites (http://www.metaboanalyst.ca/). Peak intensities were subjected to a log transformation and auto-scaling to achieve an approximately normal distribution. ANOVA module, a partial least squares-discriminant analysis module, and a heatmap module were used for data analysis and visualization.

## Results

### UFP-induced changes in the structures of gut microbial communities

**Figure 1:**
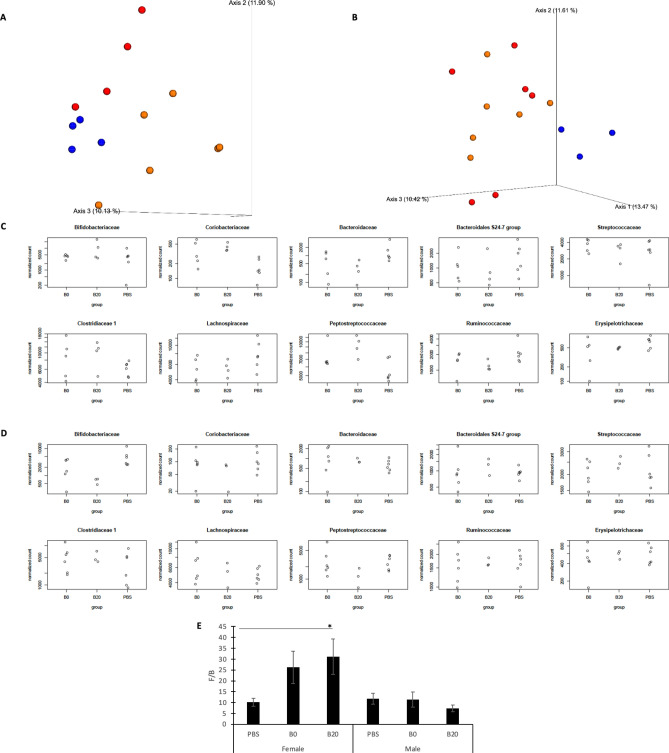
The relative abundance of bacterial levels in mice cecum samples. PCoA plots based on OTU of mice cecum samples. The results were generated based on the Jaccard distance matrix. Females (**A**) and males (**B**). The colors represent different group identities, of which red stands for B0, blue stands for B20 and orange stands for PBS group. Expression (DESeq2 normalized counts) of 10 bacterial families for PBS, B0, and B20 groups are shown for both females (**C**) and males (**D**). (**E**) The comparison of calculated ratio of *Firmicutes to Bacteroidetes* for both female and male groups. *Indicates statistical significance with *p* < 0.05.

To assess the gut microbial community structures among different samples from the treatment groups, 3D PCoA plots (Fig. 1A,B) were used to compare microbial community structure based on Jaccard distance in female and male mice. Each axis represents one of the three primary principal components (PC) and represents the OTU variation. In female groups, 17.30%, 11.90%, and 10.13% of the variation were explained by PC1, PC2, and PC3, respectively. In male groups, 13.47%, 11.61%, and 10.42% were explained by PC1, PC2, and PC3, respectively. The colors represent different group identities, of which red represents petrodiesel (B0), blue 20% soy biodiesel blend (B20), and orange the control (PBS) group. Each treatment group included six individual mice; however, due to the loss of a few mice before the end of experiment, low extracted amount of DNA, and the low DNA sequencing readings of a few samples in some of the groups, not all six samples from each group were used for statistical analysis (3 < n < 6). A close examination of the PCoA plot from Fig. 1A indicated that oral administration of the different types of UFPs to female obese mice was associated with differences in the gut microbial population compared to the PBS control group. In Figure S2A, Jaccard distance demonstrated compositional differences in the microbiome between both B0 versus PBS groups (PERMANOVA, *p* = 0.011, Table S3) and B20 versus PBS groups (PERMANOVA, *p* = 0.0051, Table S3). In contrast, males were similar to controls after B0 ingestion but B20 presented a cluster separated from the other groups (Figure S2B). As seen in Fig. 1B, the B20 group was distinct from the B0 and PBS groups, which were partially overlapped but this was not significant (PERMANOVA, *p* = 0.055, Table S3). Besides the OTU data comparison, both B0- and B20-induced gut microbiome perturbations at the gut bacterial family level were also examined for an effect of sex (illustrated in Fig. 1C for females, and Fig. 1D for males). In addition, other bioinformatics matrices, such as Bray–Curtis distance, unweighted/weighted UniFrac distance were also applied to our results to understand the differences among the three experimental treatment groups in both female (Figure S3, A–C) and male mice (Figure S3, D–F). These additional matrices again demonstrated that UFP treated mice, especially the B20 groups are having a trend of differentiating from the PBS groups. Expression (DESeq2 normalized counts) of 10 bacterial families for PBS, B0, and B20 groups for both females (Fig. 1C) and males (Fig. 1D) was plotted. A total of 10 bacterial families detected in our study were found in all of the animals (both male and female). *Coriobacteriaceae* were found to significantly increase (P_adj_ < 0.05) in female obese groups treated with UFP B0 compared to the PBS control group (Table S4a). Three of these families, including *Coriobacteriaceae*, *Bacteroidaceae*, and *Peptostreptococcace*, were found significantly changed in female obese groups treated B20 UFP compared to the PBS control group (Table S4b), while *Coriobacteriaceae* and *Peptostreptococcace* showed an increase in the B20 treatment and *Bacteroidaceae* abundance decreased in the B20 treatment. In the male obese groups, one bacterial family (*Bifidobacteriaceae*) significantly decreased in obese groups treated B20 UFP compared to the PBS control group (Table S4d).

Two bacterial divisions, the *Firmicute*s and the *Bacteroidetes*, were previously reported to dominate the distal gut microbiota from all groups (80.7% and 16.9% of all sequences, respectively) in both mice and humans^55^. Data obtained from animal models revealed consistent differences with a significant increase of the *Firmicutes* and decrease of the *Bacteroidetes* levels in obese mice, which could promote adiposity or, alternatively, indicate a host-mediated adaptive response to energy uptake/storage^56^. In the present study, Fig. 1E shows the calculated ratio of *Firmicutes to Bacteroidetes* using the relative abundance. The x-axis represents the group information and the y-axis the ratio of *Firmicutes* to *Bacteroidetes*. For the female obese groups, this ratio showed an increasing trend from 10.16 in the PBS group to 26.24 in the B0 group, and 31.14 in the B20 group. In the male obese mice groups, the F/B ratio was 11.78, 11.44, and 7.36 for PBS, B0, and B20 groups, respectively. Using the pairwise t-test, a significant difference (*p* < 0.05) between the B20 and PBS group was observed but no significant difference was seen between B0 and PBS groups in female obese mice, and the average F/B ratio of B0 was greater than PBS groups. Accumulating evidence suggests that an increase of F/B ratio and some bacterial function (*Bifidobacteria*) is related to the increase of various opportunistic pathogens and some endotoxins-producing Gram-negative bacteria^57–59^. The results from this study suggest that exposure to UFPs, especially B20, alters the gut bacterial community and may promote adverse health effects in the female host only. Notably, no significant differences in responses to UFP were found among male obese mice groups. It is also worth noting that in recent years, the validity of using the F/B ratio as a gut dysbiosis biomarker in the obese population has been debated in many contradictory studies^60,61^, and such discrepancies might be due to a variety of difference in data collection methods, data interpretation strategies, and poor characterizations of other phenotypic factors from study participants. Therefore, while we present these data here, we also acknowledge that the F/B ratio may not be indicative of a direct link to health status in obese individuals.

### Distinguishing features for differentiating responses to UFPs treatments compared to control

**Figure 2:**
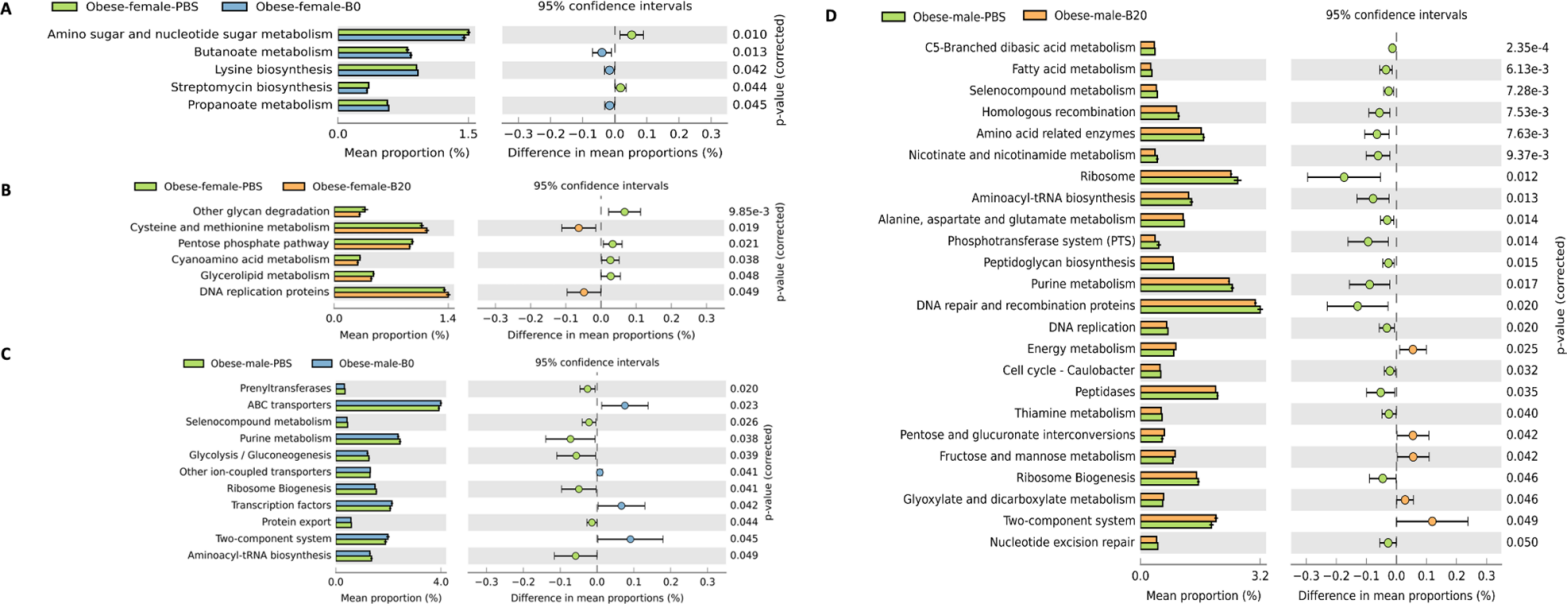
The difference in mean proportion of microbial function between (**A**) female PBS and B0; (**B**) female PBS and B20; (**C**) male PBS and B0 and (**D**) male PBS and B20 along with the associated 95% confidence interval of this effect size and the *p* value less than 0.05.

To further analyze the functional profiles of microbial communities, the functional composition of the metagenome data collected in this study was predicted by PICRUST (phylogenetic investigation of communities by reconstruction of unobserved states) using Greengenes reference databases. The known bacterial gene counts obtained through the integrated microbial genomes database and comparative analysis system were used to predict the gene content of a given metagenomic sample^62^. Welch’s t-test with a *p* value of less than 0.05 was used for statistical analysis*.* Gene-functional data were input into the STAMP software package. Figure 2 shows the level 3 functional categories of PBS-treated obese mice that were significantly (*p* < 0.05) different from either B0 or B20 groups. Within each sex, the B0, B20, and PBS treatments are illustrated in blue, orange, and green, respectively. The 95% confidence level is shown as a dashed line in the right part of the plot, and the enrichment of each functional category is shown as a circle of the corresponding color. The *p value* of each functional category is listed as a column on the right next to the 95% confidence level. Consistent with the changes in the gut microbiome community structures, the mean proportion of microbial function between PBS control groups and treatment groups was also significantly different (*p* < 0.05), where many of the key metabolic pathways were identified as altered by UFPs. It is interesting to note that although the microbial communities may be altered readily in female obese mice post-UFP exposure compared to male obese mice, based on this analysis, many of the predicted metabolic functional alterations were also observed in the male obese mice post-UFP exposure. This observation emphasizes the importance of analyzing the gut microbial metabolic features together with genomic information.

### UFP-induced changes in the metabolic profile of the gut microbiome

**Figure 3:**
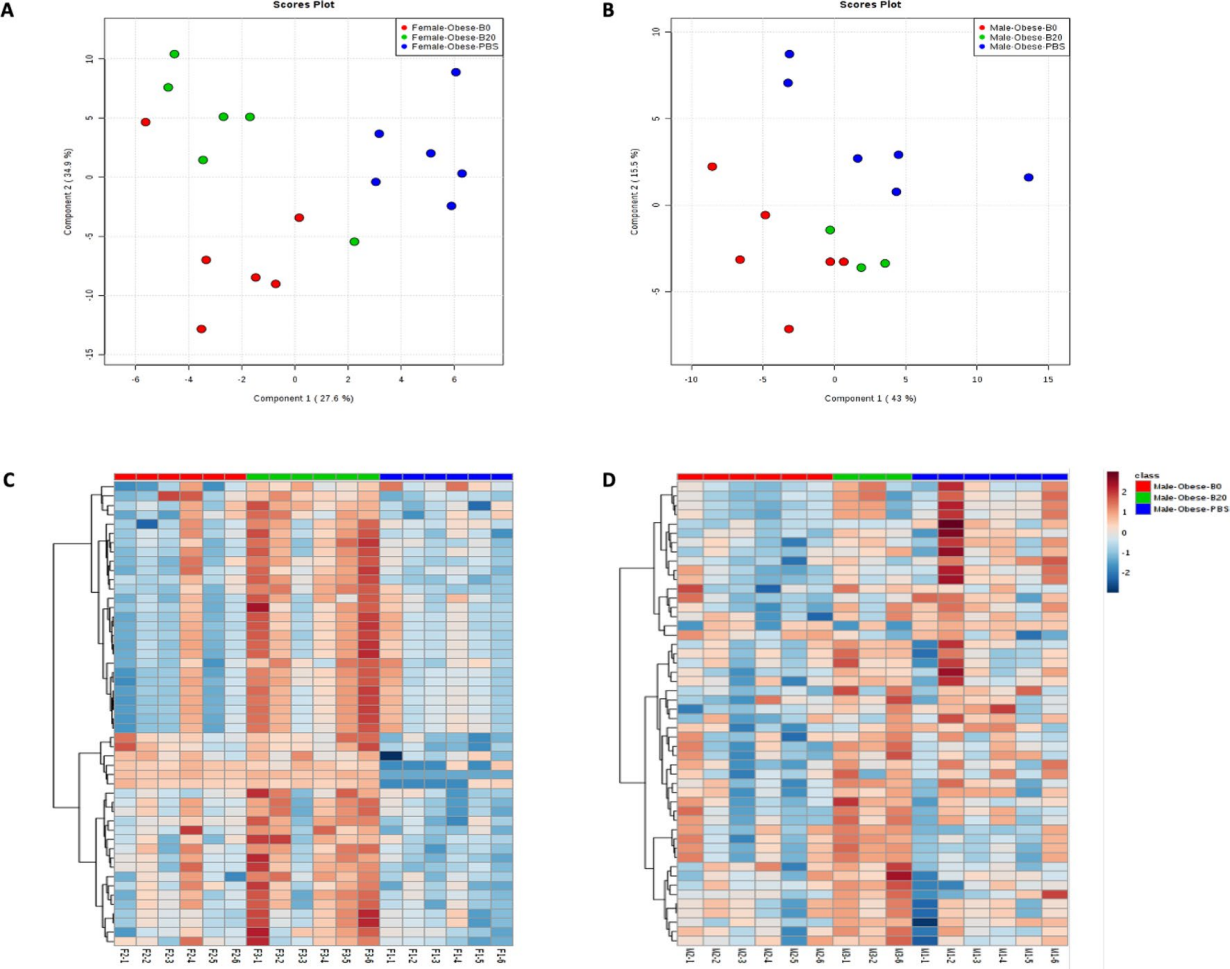
Gut microbial metabolic profiling of female and male mice based on the metabolomic analysis of cecum samples. Within each sex, the B0, B20, and PBS treatments were illustrated in blue, orange, and green. (**A**, **C**) showed females PLS-DA plots and heatmap while (**B**, **D**) showed the results from the male groups. The color within each heatmap indicates the expression level of each metabolite, dark red indicates the highest abundance and dark blue indicates the lowest abundance of the metabolite in the sample.

After analyzing and predicting the genomic functions via the 16S rRNA gene sequencing data, we further characterized the metabolome of both the gut microbes and the host. Our targeted metabolomics analysis results show that the UFP administration perturbed the metabolic profiles of the gut microbiome in both female and male obese mice, and the overall metabolic profiles of mice from the different treatment groups are shown in Fig. 3. A total of 135 targeted polar metabolites were detected among all cecal samples in both sexes. As shown in Fig. 3, the metabolic profiles of UFP-treated groups in both females and male obese mice can be differentiated from the PBS control groups based on the different metabolite intensities detected via the metabolomics approach, and the overall profile can be largely separated by using the first two components of PLS-DA results (Fig. 3A,B). If we take a step further and remove the PBS groups, as they were clearly different from the UFPs treatment groups, distinguishing group separation can also be observed when only comparing the B0 and B20 groups (Figure S4a and S4b). Figure 3C,D show the metabolic profile (top 75 based on ANOVA) heatmap of mice cecal samples as females shown in Fig. 3C and males shown in Fig. 3D. The horizontal axis represents the group information, while the vertical axis represents individual metabolites. The color indicates the expression level of each metabolite, with dark red as the highest abundance and dark blue the lowest abundance of the metabolite in the sample. The heat map analysis applied the top 75 metabolites that differed significantly (ANOVA *p* < 0.05) to make the heat map more readable. The result reveals that the metabolic profiles of females were generally clustered within their treatment groups, whereas differences between groups were more difficult to discern in males.

**Figure 4:**
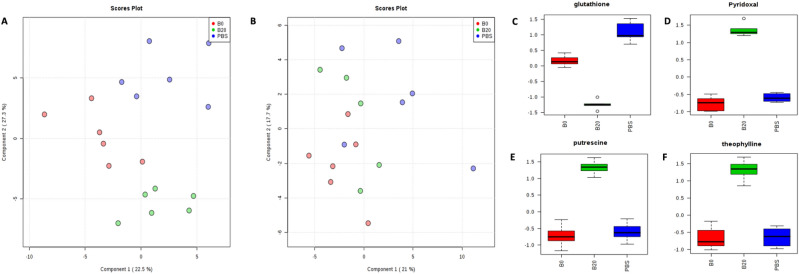
Host metabolic profile difference in both female and male mice and the most contributed metabolites in plasma samples of female mice. The PLS-DA plots showed the metabolic profile of B0, B20, and PBS groups for females (**A**) and males (**B**). Metabolites that contributed most to the significantly different clustering of female mice based on their *p* values were shown in (**C**–**F**).

The host metabolic profile was also assessed by the detection of plasma metabolites through our targeted metabolomics platform. As shown in Fig. 4A,B, the PLS-DA plots indicated good differentiation on the metabolic profiling of female obese mice from the two UFPs treatment groups and the PBS control, while less distinctive groups were observed in male obese mice. These results were consistent with the mice gut microbiome metabolites data, which collectively showed less alteration in males in terms of both host and gut microbiome metabolic profiles post-UFPs exposure. The clear differentiation among female groups was supported by several strongly differentially expressed metabolites after UFPs exposure. The top four metabolites that contributed to the clustering (based on ANOVA) are shown in Fig. 4C–F. Clearly, after exposure to UFPs, especially those from B20 combustion, glutathione was significantly (*p* < 0.05) decreased in the plasma of female mice, while pyridoxal, putrescine, and theophylline increased significantly in female hosts, but no significant changes were observed for B0 groups, relative to the PBS control. Of interest is the metabolite, theophylline, a drug derived from the methylxanthine, a purine derivative. Further investigation will be needed to elucidate the reproducibility of these findings and the potential biological consequences. Although a fewer number of differential metabolites among the three male groups were observed compared to females, some representative host metabolites were still altered significantly. For instance, myo-inositol, a carbocyclic sugar that mediates cell signal transduction in response to a variety of compounds (e.g. hormones, neurotransmitters, growth factors, etc.) was significantly decreased in B20 treatment groups compared to PBS control groups in male plasma (*p* = 7.85E−5) (Figure S5). Again further investigation of the biological consequences will be needed.

### UFP-induced metabolic pathway alteration

**Figure 5:**
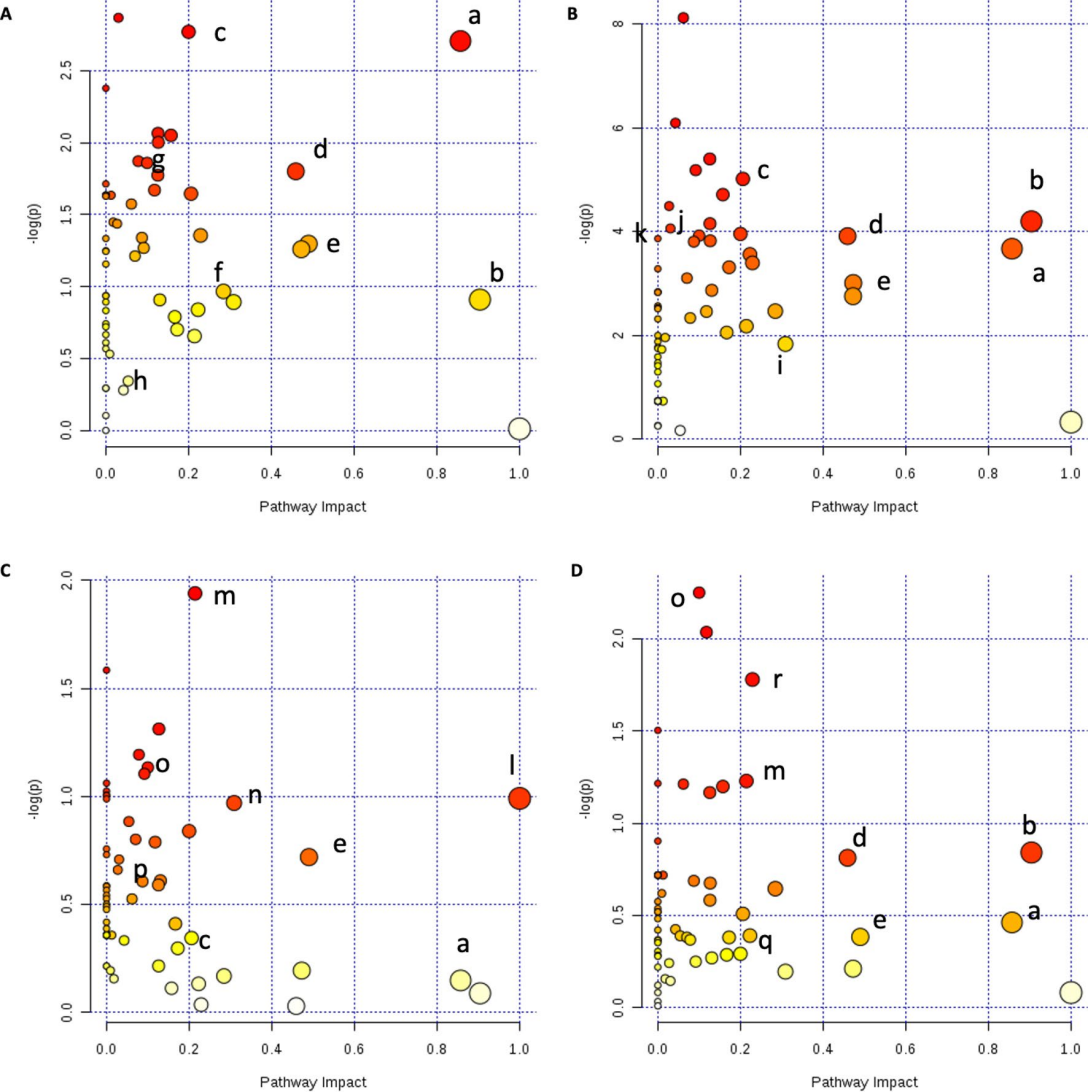
Gut microbial metabolic pathway analysis on cecum samples. Female PBS versus B0 (**A**); female PBS versus B20 (**B**); male PBS versus B0 (**C**) and male PBS versus B20 (**D**). Some representative metabolic pathways were assigned arbitrary lower case letters in alphabetical order and the full names are shown in Table S1.

**Figure 6:**
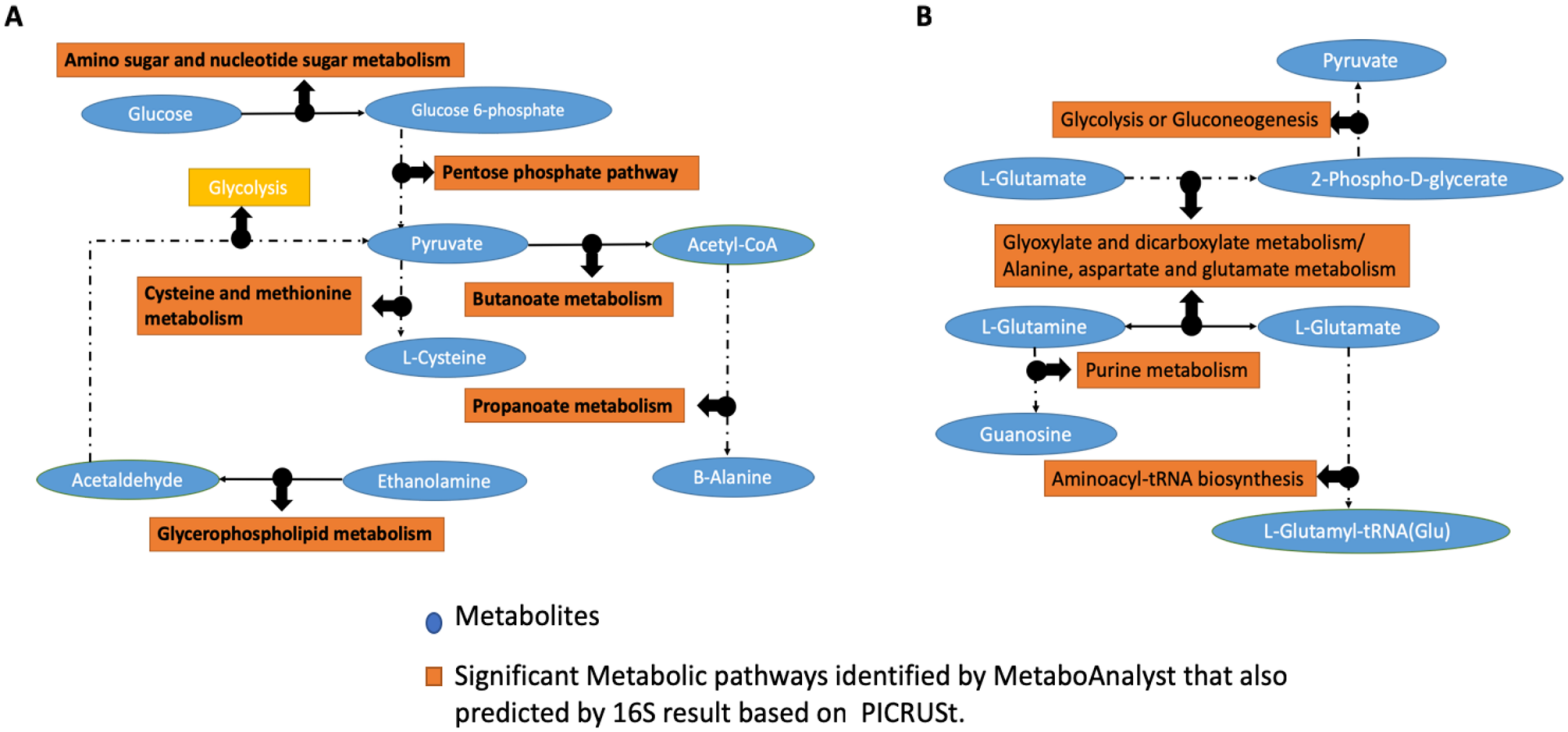
Connections among metabolic pathways that matched with microbial functional prediction result. Females (**A**) and males (**B**).

**Figure 7:**
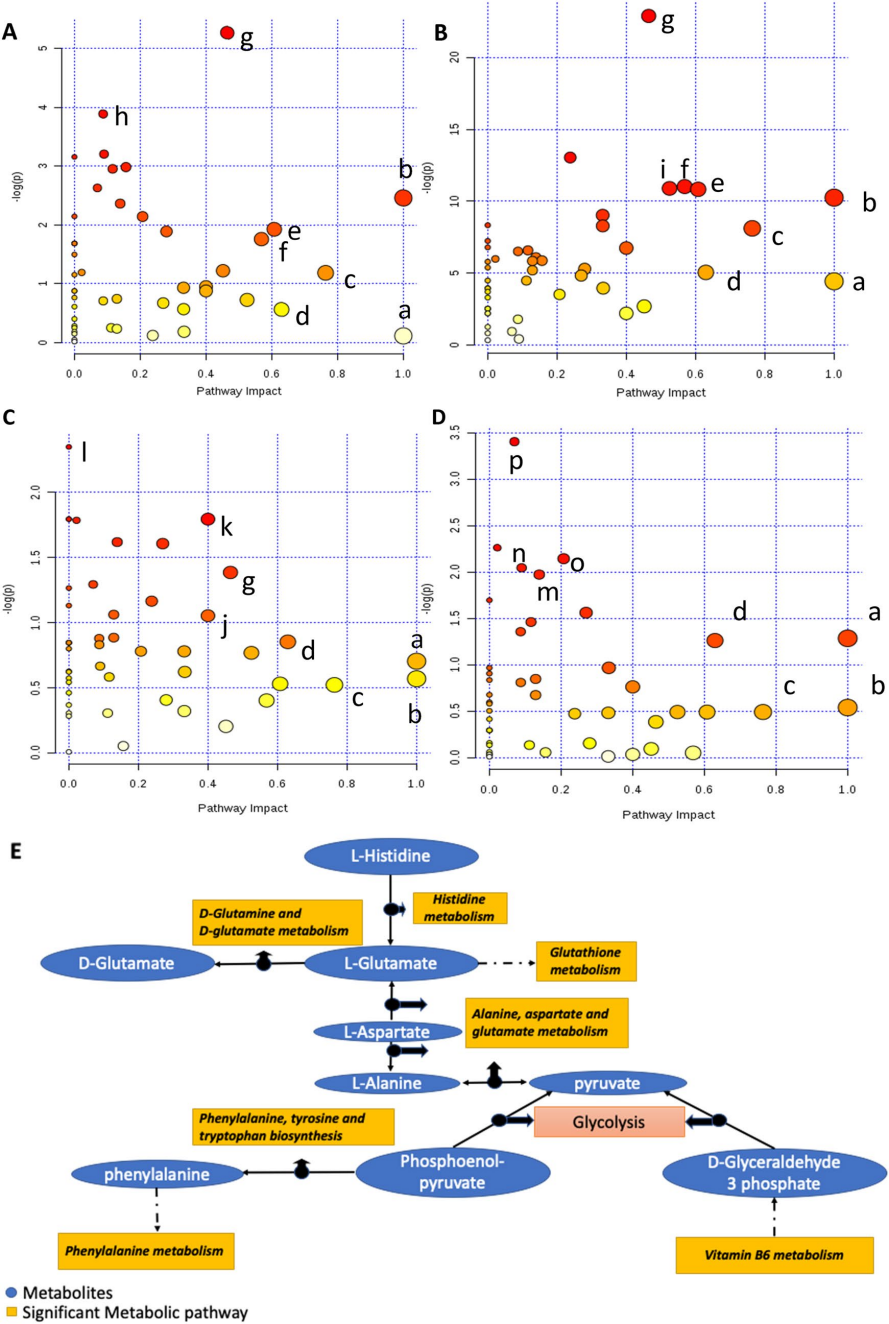
Host metabolism pathway analysis on plasma samples. Female PBS versus B0 (**A**); female PBS versus B20 (**B**); male PBS versus B0 (**C**) and male PBS versus B20 (**D**). Detailed metabolic pathways information is listed in Table S2. Significantly altered metabolites and their pathways were summarized in the pathway tree plot (**E**).

To fit all the metabolites detected into the context of a connected metabolic pathway network, we conducted metabolic pathway impact analyses to put individual metabolites in a broader aspect of the biochemical network. All detected metabolites of each sex were included in the metabolic pathway analysis, so the broader coverage of extensive metabolic networks could be achieved. Figure 5 shows the major metabolic pathways of gut microbial bacteria that were impacted by UFPs exposure. The x-axis is the metabolic pathway impact value (from pathway topology analysis, which is calculated as the sum of the importance measures of the matched metabolites normalized by the sum of the importance measures of all metabolites in each pathway), while the y-axis is the statistical significance (represented by log *p* value) of the impacted pathway from exposure groups compared to the control group. The dot size corresponds to the pathway impact value and the dot color corresponds to the *p value*. It is interesting to note that metabolic pathways, such as alanine, aspartate, and glutamate metabolism, glycolysis or gluconeogenesis as well as butanoate metabolism which are marked as metabolic pathways b, c, and f in Fig. 5, respectively, were also found as matched with the functional prediction results from Fig. 2 and were identified as significantly (*p* < 0.05) impacted metabolic pathways. All of the metabolic pathways that were significantly (*p* < 0.05) changed in pathway analyses of cecal microbial bacteria, as well as matched to functional prediction analyses, were aligned as shown in Fig. 6A for males and Fig. 6B for females; solid arrows indicate a direct connection among metabolic pathways and metabolites while dashed arrows indicate indirect connections (multiple intermediate steps were excluded to save space). Host metabolic pathways analyses were also conducted. As shown in Fig. 7A–D, pathway analyses showed the important metabolic pathways impacted by UFPs administration. Pairwise comparison of female PBS versus B0 (Fig. 7A); female PBS versus B20 (Fig. 7B); male PBS versus B0 (Fig. 7C) and male PBS versus B20 (Fig. 7D) were conducted. Interestingly, all four analyses had similar metabolic pathways that were most impacted. Detailed metabolic pathways information is listed in Table S2, as metabolic pathways in bold font were significantly (*p* < 0.05) altered pathways appearing repetitively in female and male groups after UFPs exposure. An overview pathway connection plot was generated based on these major impacted metabolic pathways among PBS control and UFPs treatment groups (Fig. 7E). Several amino acid metabolic pathways, together with energy metabolic pathways (e.g., glycolysis) were the primary pathways impacted by UFPs exposure during the comparison of the host metabolism.

## Discussion

Ultrafine particles from multiple sources are ubiquitous in indoor air, ambient urban air, and the diet and may contribute to many respiratory and cardiovascular effects^63^. After oral ingestion, the surfaces of ultrafine particles are likely to become modified^63^ and the modified surface may adsorb biomolecules readily^64^. The adsorbed biomolecules can then be taken up by the intestinal cells and may impact the function of the gastrointestinal tract^29^. Previous studies have shown associations between traffic density and obesity^65^, but limited reports have been focused on the link between effects of fuel-type specific UFPs exposure and holistic metabolic dysregulation in the obese of both sexes. Moreover, previous studies seldom focused on the effects of UFP exposure on the gastrointestinal (GI) tract for both gut microbiome and host metabolism, and an investigation of the large-scale metabolic changes during UFP perturbation to the gut is still lacking. In the present work, we provide the first evidence that UFP exposure via the GI tract can alter the metabolic profiles in the host as well as the gut microbiota community in the distal intestine, and the findings suggest new insights into the relationship between air pollution and the gastrointestinal responses of obese rodents. When evaluating the gut microbial community after exposure to the two different types of UFPs, the PCoA of 16S analysis results enabled us to visualize that UFP treatment groups tended to form a cluster distinctly separate from the PBS control samples in female groups, and partial separation was achieved in male group comparisons (Fig. 1). These results suggested that UFPs-induced gut microbial changes might be sex-specific. It is also noticed that not all matrices are providing clear separations for three treatment groups in female or male mice, and we attributed the lack of clear boundaries in these cases to the complex data matrix from 16S results and the relatively small group size in this study. Additional experiments may be warranted in the near future to provide validations to our findings. In addition, at the bacterial phylum level, *Firmicutes, Bacteroidetes, and Actinobacteria* were the major phyla identified that were impacted by the exposure. As in humans, *Firmicutes* and *Bacteroidetes* are the most abundant bacterial phyla in these study animals, whereas *Actinobacteria* is present at lower abundance in mice^56^. Upon the oral exposure to the UFP treatments, a slight decrease in the relative abundance of both *Firmicutes* and *Bacteroidetes* in obese female mice was observed. Several studies have reported that a decrease in the abundance of several taxa within the *Firmicutes* phylum is related to structural imbalances and dysbiosis that occur in inflammatory bowel disease^66^. A significant decrease in the abundance of *Bacteroidetes* in germ-free mice colonized with human microbiota was reported upon a dietary shift to a high-fat, high-sugar “Western” diet^67^. However, the same level of change in the relative abundance of both *Firmicutes* and *Bacteroidetes* was not found in the male mouse obesity model used in this study. Despite the reduction in the abundance of both *Firmicutes* and *Bacteroidetes* after exposure to UFPs, there was a significant increase in the *Firmicutes* to *Bacteroidetes* ratio in the female B20 treatment group*.* In previous studies, the *Firmicutes/Bacteroidetes* ratio has been reported to be positively correlated with the obese phenotype, independent of diet^56^. Based on our results, a disadvantageous shift in gut microbial communities may occur as a consequence of UFP exposure in female, but not male mice. This is consistent with previous findings that sex-specific effects on the gut microbiome may occur when animals were exposed to the pesticide, organophosphate diazinon^68^.

Several distinguishing features of the biological functions of the microbiota associated with exposure to UFPs can be identified in this study. There were eleven gut microbial functional pathways that contributed a large fraction of total significant function counts among the female obese mice. Reduced functions of gut microbes in the B0-exposed female mice included amino acid, sugar, and nucleotide sugar metabolism, and streptomycin biosynthesis while butanoate metabolism, propanoate metabolism, and lysine biosynthesis were enriched in these samples in comparison to the same-sex PBS control samples. In addition, other enriched functions included cysteine and methionine metabolism, and DNA replication proteins that were associated with the exposure to B20 compared to the PBS control in females. Other functions such as glycan degradations, pentose phosphate pathway, cyanoamino acid metabolism, and glycerolipid metabolism were reduced after B20 exposure in female obese mice. As reported previously, the pentose phosphate pathway (PPP) may regulate the production of NADPH, which can play vital roles in several biological processes such as nucleic acid synthesis and DNA replication^69^, reductive biosyntheses, and in the protection of cells from reactive oxygen species (ROS)^70^, which indicated the potential biological impact of B20 UFPs to many biological functions. In the screening of hub genes and pathways in colorectal cancer with microarray technology, butanoate metabolism, and DNA replication were included in the 34 functions that were significantly over-represented in colorectal cancer^71^. Among the 34 functions, DNA replication was the most significantly over-represented pathway. The investigators of this prior study further explored interactions within the colorectal cancer-related pathways, ranking the P53 signaling pathway, cytokine-cytokine receptor interaction, and type I diabetes mellitus as the three most important interactions^71^. Therefore, we speculate that frequent oral exposure to UFPs in female obese mice may be associated with a higher risk for colorectal cancer development later in life. On the other hand, twenty-six functional pathways contributed significant counts among male samples (Fig. 2). Only one of these functions, DNA replication, was also significantly expressed in female samples.

Intestinal microbiota metabolites are important to host-microbe interactions and consequently, are key determinants of the health or disease of the intestinal tract^72^. Gut microbiota varies in populations and is regulated by factors that regulate host metabolism, immunity and inflammatory responses^73–75^. In addition, gut microbial metabolites also play an important role in controlling the gut microbiota population and diversity. For example, cholesterol is produced in the intestine and converted to bile acids; and this process is critical for cholesterol homeostasis^76^ and the prevention of the accumulation of toxic metabolites in the liver and other organs^77^. In this study, the results showed an obvious perturbation in gut metabolic profiles of female obese mice compared to males after the administration of UFPs that were collected from both B0 and B20 combustion. For example, females showed a significant increase (*p* < 0.05) in the pyridoxal levels in both intestinal and plasma samples (Figure S6). It has been shown that increased levels of pyridoxal were associated with a variety of inflammatory disease conditions which include rheumatoid arthritis, inflammatory bowel disease, and cardiovascular disease^78^. Allantoin, another representative metabolite, showed greater abundance after both UFP treatments in females but was undetectable in the female PBS control group. A previous study reported that this metabolite can mitigate inflammation by inducing the reduction in the levels of IL(interleukin)-4 and IL-5^79^. However, a sex-specific difference existed, as insignificant differences in both intestinal microbiome and plasma were observed when comparing UFPs treatments to the PBS control group in obese males in our study. This may be related to the similar metabolic profiles between the male B0 treatment and the male PBS treatment, or the large relative standard deviation (RSD%) of some metabolic measurements in the male B0 samples. When we compared the male B20 groups and the male PBS groups, statistically significant perturbations in metabolic profiles were observed in plasma. For example, myo-inositol, the most significantly changed metabolite in the host metabolic comparison, was significantly decreased in B20 treatment groups compared to PBS control groups in male plasma (Figure S5). The decreasing level of myo-inositol can be associated with inflammation induced by UFPs^80,81^. Consistent with the F/B ratio result from the gut microbes, the results of the host metabolite comparison also indicated a higher impact of ingested UFPs in the gastrointestinal tract in females than males. It is also acknowledged that due to the limit blood smaple volumes available, we were unable to perform circulating metabolic phenotypes such as blood glucose and inflammatory markers, which should better assist the data interpretation and further understanding of the impact of UFP to obesity. Future study will be planned to conduct these measurements in addition to gut microbiome and metabolite analysis.

## Conclusion

In this study, we combined metabolomics and 16S rRNA gene sequencing techniques to study the effect of oral exposure to ultrafine particles from the combustion of different diesel fuels on the gut microbial community and their metabolic processes in an obese mice model. The results showed that the gut microbiome composition, as well as their metabolic profiles, were altered after direct exposure to two different types of ultrafine particles in the intestinal tract of both C57BL6 male and female mice. Our genomic results predicted the possible functional impacts of ultrafine particles on the mouse gut microbiome, and our metabolomics analysis validated some of the functional predictions, and both genomic and metabolomics results suggested a difference by exposure of obese mice to B0 and B20 UFPs. Interestingly, different levels of perturbation on the gut microbiome were observed between females and males, and stronger perturbation effects on females were observed based on data from both 16S rRNA gene sequencing and metabolic profiling, suggesting female obese mice may be susceptible to a higher risk of metabolic dysfunction during UFP exposure. These results could decipher the role of the gut microbiome in modulating the gastrointestinal system and the host metabolic process in response to UPF ingestion from environmental exposure. The documented changes of the gut microbiome and metabolic profile post-UFP ingestion in this obese mice model could serve as useful preliminary data to the future investigation of the potential sex-specific health effects of UFPs exposure in humans, and the perturbation caused by different sources of UFPs may also contribute to the understanding of biological effects of UFP ingestion from different fuel types.

## Supplementary Information

Supplementary Informations.
