# Supplementary material for: Ultrafine particles altered gut microbial population and metabolic profiles in a sex-specific manner in an obese mouse model
Source: Sci Rep. 2021 Mar 25;11:6906. doi: 10.1038/s41598-021-85784-4 (PMC7994449; doi:10.1038/s41598-021-85784-4)

# *Supporting Information:*

**Ultrafine Particles Altered Gut Microbial Population and Metabolic Profiles in a Sex-Specific Manner in an Obese Mouse Model**

Kundi Yang^1#^, Mengyang Xu^1#^, Jingyi Cao^1^, Qi Zhu^2^, Monica Rahman^1^_,_ Britt A. Holmén^3^, Naomi K. Fukagawa^4^, and Jiangjiang Zhu ^5, 6^*

1. Department of Chemistry and Biochemistry, Miami University, Oxford, OH, 45056

2. Department of Biology, Miami University, Oxford, OH, 45056

3. School of Engineering, University of Vermont, Burlington, VT 05405

4. USDA ARS Beltsville Human Nutrition Research Center, Beltsville, MD 20705 USA

5. Department of Human Sciences, The Ohio State University, Columbus, OH, 43210

6. James Comprehensive Cancer Center, The Ohio State University, Columbus, OH 43210

# These two authors contributed equally to this work

* Corresponding author,

Email: zhu.2484@osu.edu

Tel: 614-685-2226

**Figure legends**

**Figure S1**. The rarefaction curve of gut microbiota in mice cecum samples for PBS, B0, and B20 groups.

**Figure S2.** Permutation multivariate analysis PERMANOVA test on Jaccard distance of gut microbial community in different groups for both females(A) and males (B). Boxplots show distances between each group.

**Figure S3**. Beta diversity analysis of tested mice groups in our study using different bioinformatics metrices. The colors represent different group identities, of which red stands for B0, blue stands for B20 and orange stands for PBS group. (A). Bray-Curtis distance-based analysis of female groups; (B). unweighted UniFrac distance-based analysis of female groups; (C). weighted UniFrac distance-based analysis of female groups; (D). Bray-Curtis distance-based analysis of male groups; (E). unweighted UniFrac distance-based analysis of male groups; (F). weighted UniFrac distance-based analysis of male groups;

**Figure S4**. Metabolic profile-based PLS-DA plot of B0 vs B20 in (a) female obese mice and (b) male obese mice.

**Figure S5**. Plasma Myo-inositol detected from male obese mice. The box and whisker plots summarize the normalized values.

**Figure S6**. Intestinal and plasma pyridoxal levels detected from female obese mice. (a) cecum data and (b) plasma data. The box and whisker plots summarize the normalized values.


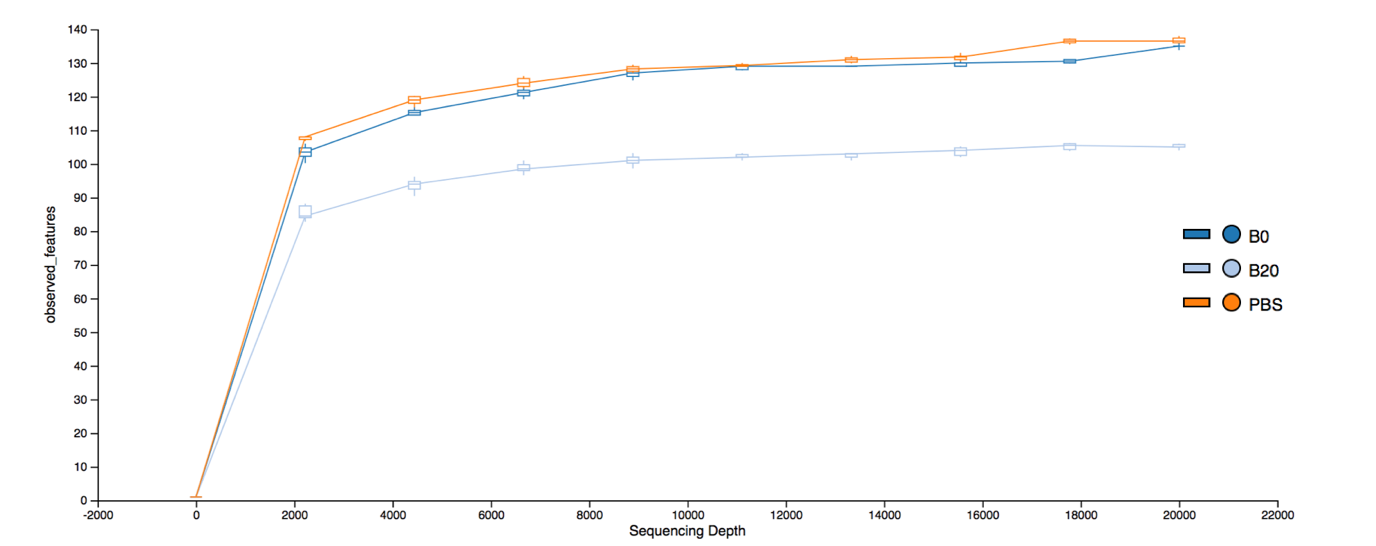


Figure S1
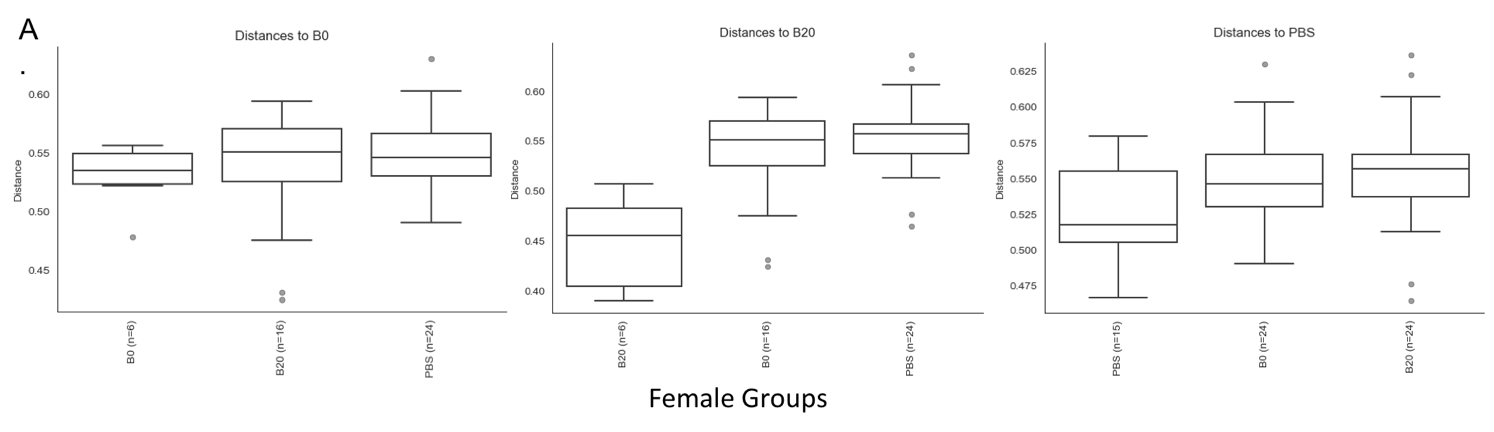


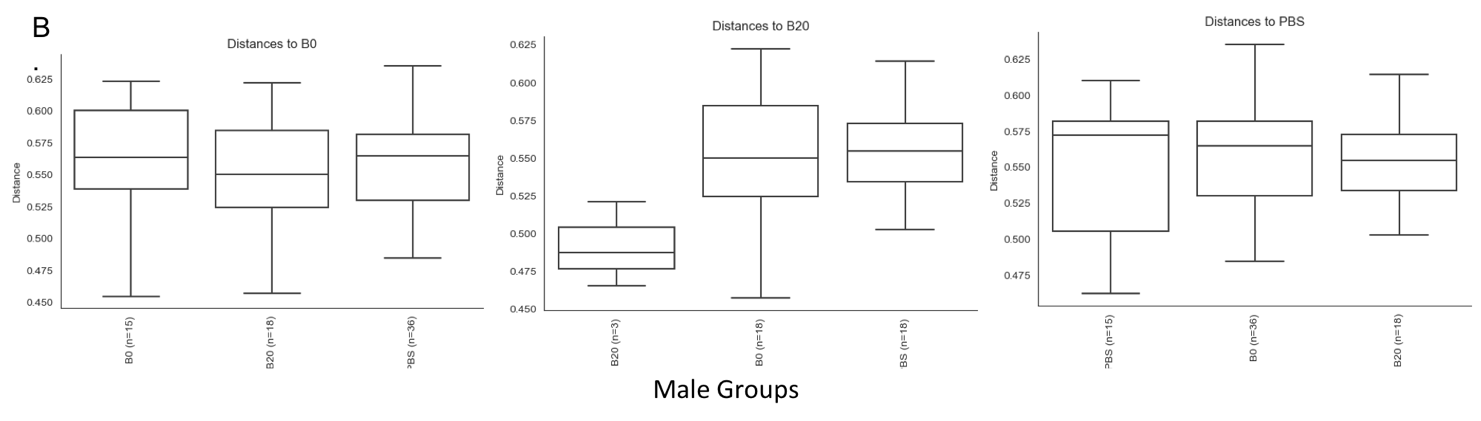


Figure S2


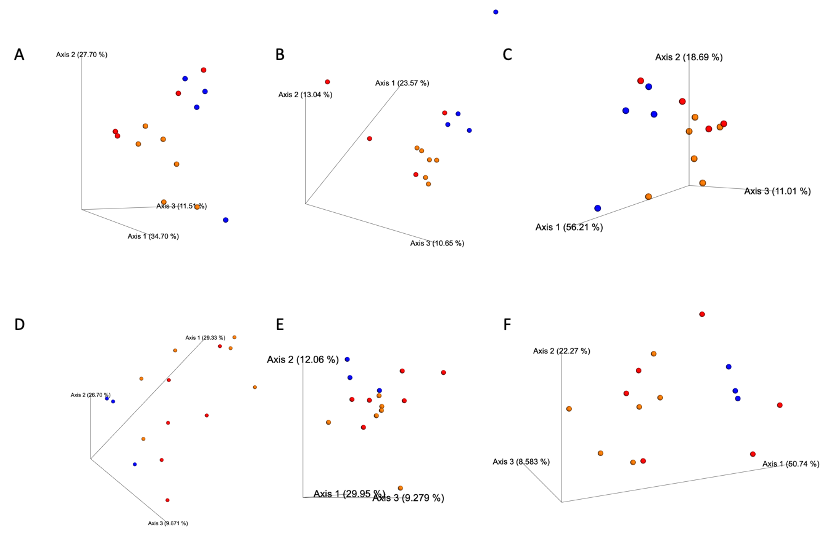


Figure S3


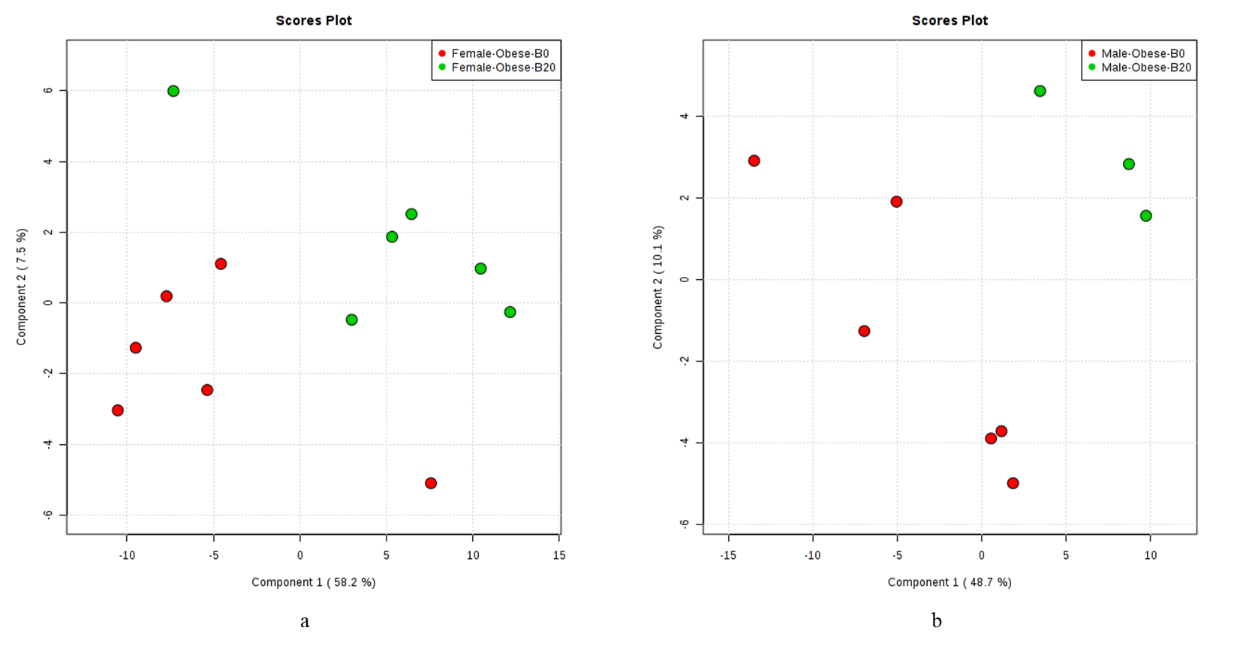
Figure S4

Figure S5


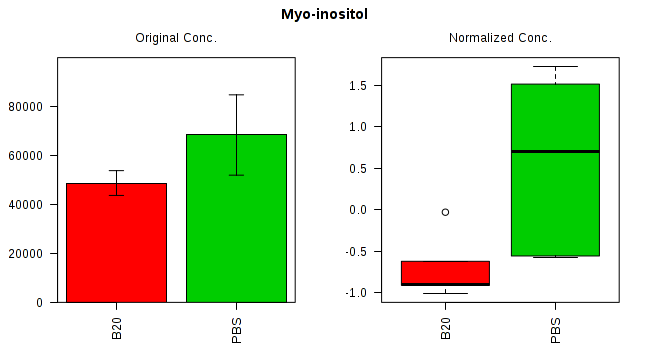

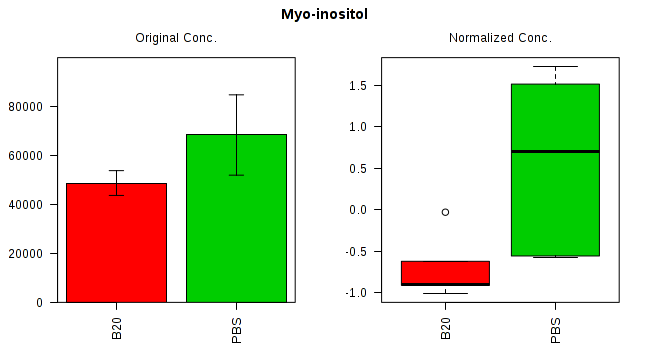


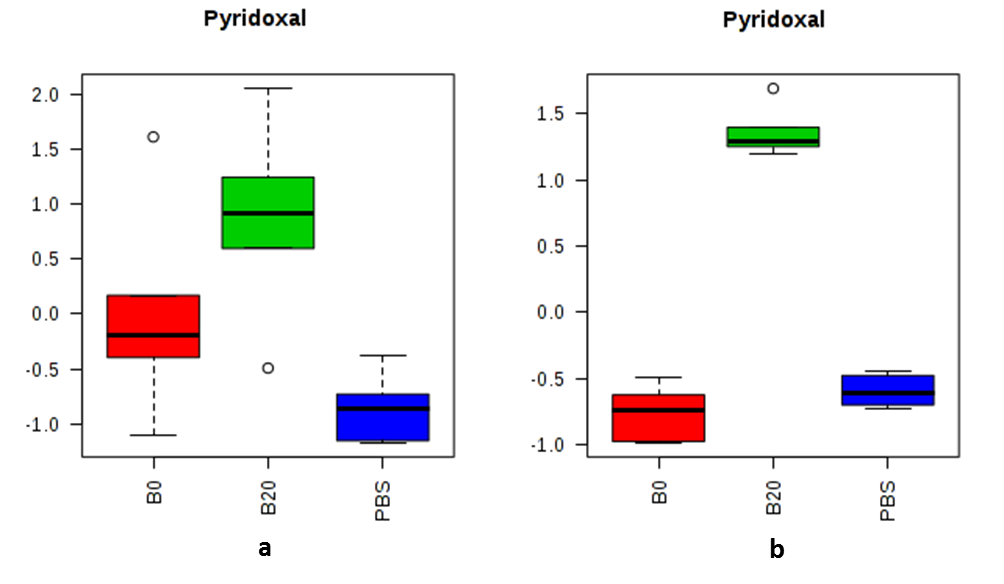
Figure S6

**Table S1**. Mice cecum microbial metabolic pathways identified in Figure 5. Pathways in bold were identified as they matched to function prediction at genus level (Figure 2).

| Corresponding letters in Figure 5 | Pathways |
| --- | --- |
| a | Taurine and hypotaurine metabolism |
| b | **Alanine, aspartate and glutamate metabolism** |
| c | **Glycolysis or Gluconeogenesis** |
| d | Pyruvate metabolism |
| e | Arginine and proline metabolism |
| f | **Butanoate metabolism** |
| g | **Amino sugar and nucleotide sugar metabolism** |
| h | **Propanoate metabolism** |
| i | **Cysteine and methionine metabolism** |
| j | **Glycerophospholipid metabolism** |
| k | **Pentose phosphate pathway** |
| l | Inositol phosphate metabolism |
| m | Biotin metabolism |
| n | Cysteine and methionine metabolism |
| o | **Purine metabolism** |
| p | **Aminoacyl-tRNA biosynthesis** |
| q | **Glyoxylate and dicarboxylate metabolism** |
| r | Citrate cycle (TCA cycle) |

**Table S2**. Host metabolic pathways (using plasma metabolites) identified in pathway analysis (Figure 7). Pathways in bold were identified as they were significantly altered pathways repeated in both female and male groups.

| Corresponding letters in Figure 7 | Pathway |
| --- | --- |
| a | **Phenylalanine, tyrosine and tryptophan biosynthesis** |
| b | **D-Glutamine and D-glutamate metabolism** |
| c | **Alanine, aspartate and glutamate metabolism** |
| d | **Phenylalanine metabolism** |
| e | **Histidine metabolism** |
| f | **Vitamin B6 metabolism** |
| g | **Glutathione metabolism** |
| h | Pyrimidine metabolism |
| i | Arginine and proline metabolism |
| j | Thiamine metabolism |
| k | Methane metabolism |
| l | Riboflavin metabolism |
| m | Glycolysis or Gluconeogenesis |
| n | Amino sugar and nucleotide sugar metabolism |
| o | Starch and sucrose metabolism |
| p | Pentose phosphate pathway |

**Table S3.** Permutation multivariate analysis PERMANOVA test on Jaccard distance of gut microbial community in different groups for both females and males.

|  | Group 1 | Group 2 | Sample size | Permutations | pseudo-F | p-value | q-value |
| --- | --- | --- | --- | --- | --- | --- | --- |
| Female | B0 | PBS | 10 | 999 | 1.392 | 0.011 | 0.0165 |
|  | B20 | PBS | 10 | 999 | 2.197 | 0.005 | 0.015 |
| Male | B0 | PBS | 12 | 999 | 1.079 | 0.332 | 0.332 |
|  | B20 | PBS | 9 | 999 | 1.423 | 0.055 | 0.1455 |

Table S4. Differential abundance of 10 bacterial families between PBS, B0, and B20 groups for both females and males by DESeq2 in R (A. Female, PBS vs. B0; B. Female, PBS vs. B20; C. Male, PBS vs. B0; D. Male, PBS vs. B20).


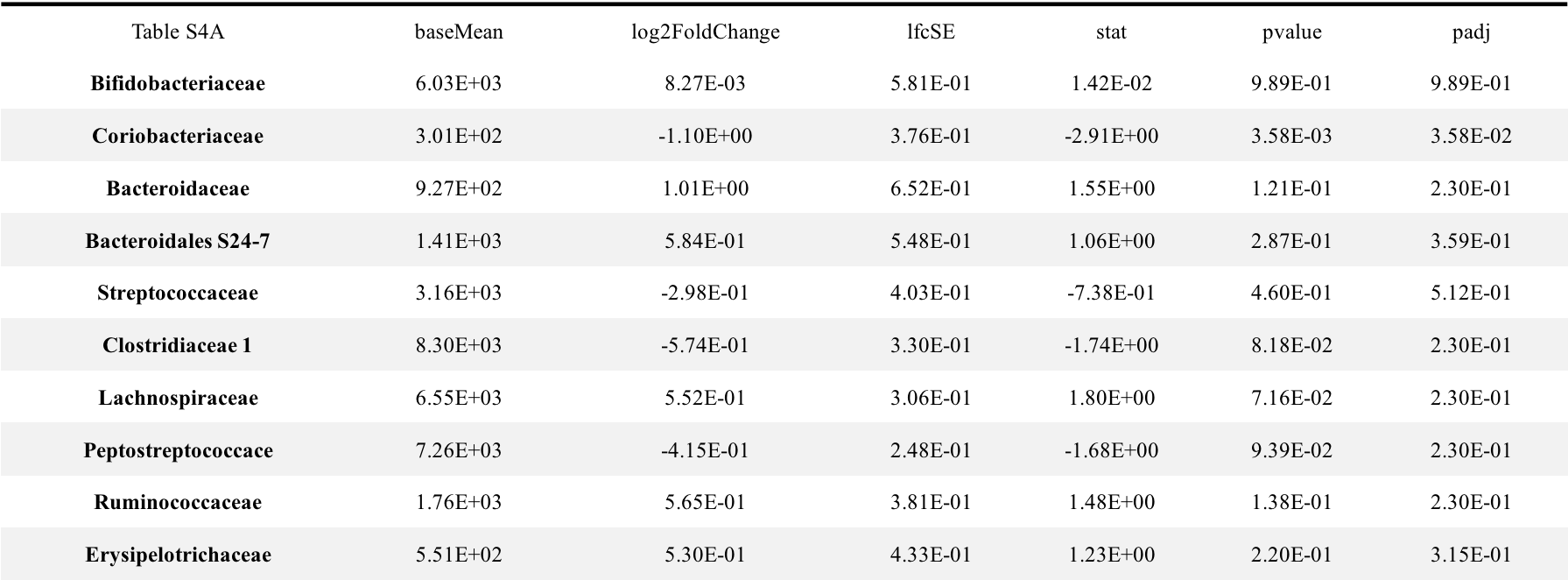


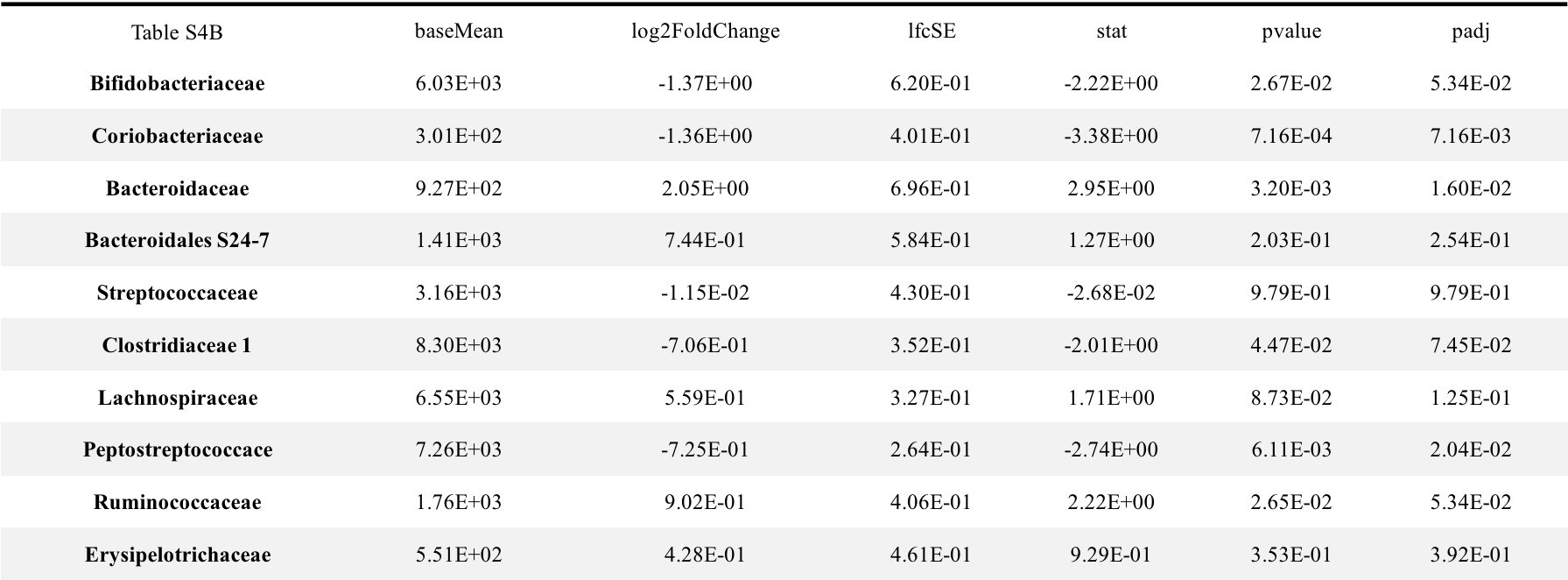


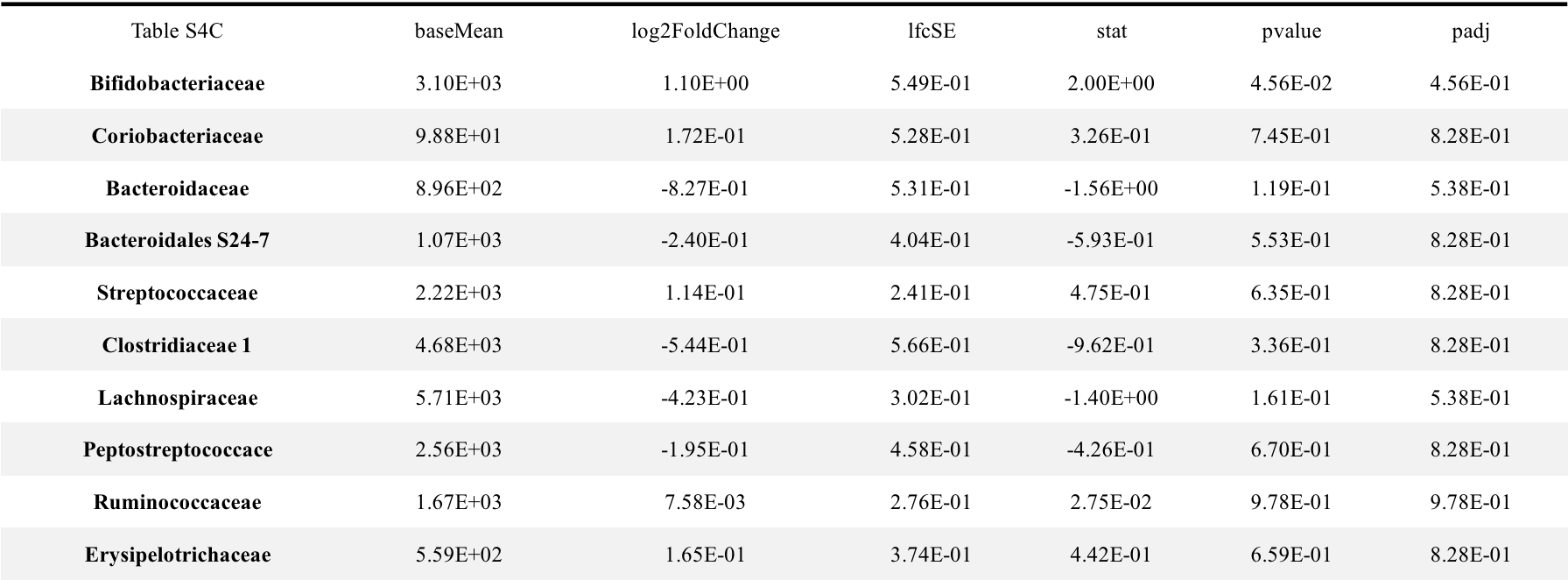


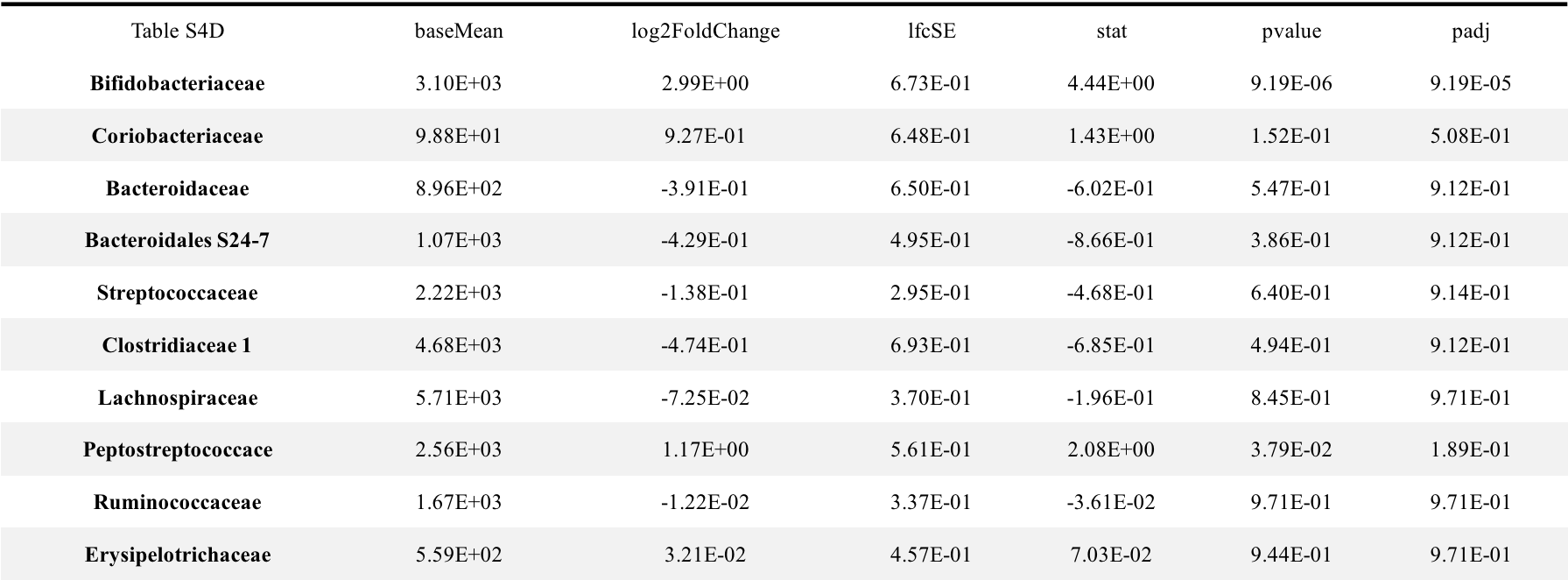

Supplement: Supplementary file 1 — Supplementary Informations. [file 41598_2021_85784_MOESM1_ESM.docx]
